# Nekrosoziac or Embalming

**Published:** 1868-09

**Authors:** 


### Nekrosoziac or Embalming.

A new mode of embalming has been introduced in New York, and is thus described:

Over two hundred members of the medical faculty assembled on Friday, April 24th, in the anatomical museum attached to the Bellevue Hospital, to be present at an autopsy of the body of a female, aged about 30, dead 76 days, which had been preserved in life-like freshness by a new process of embalming called "nekrosoziac." Prof. Doremus presided over the autopsy, in which several of the most distinguished physicians and surgeons of the city participated. This new process of embalming consists simply in a wash of the deceased body without wound or incision. Some of its specialties are to dispense with the old system, disemboweling and extracting the brain; also avoiding mutilation or injection of any kind, and acting as a thorough disinfectant. The body operated upon this day was not in the slightest degree discolored; the features were as in life, and the smell as inoffensive as of a body twenty-four hours after death. The opening of the body revealed the fact that the bowels and brains, as well as the flesh, were free of the slightest appearance of taint or of smell. The limbs were as pliable as in life. Several of the surgeons spoke in high terms of the extraordinary discovery as likely to work a revolution in the preservation and transportation of dead bodies. Another body preserved by the same process for one hundred and three days has been subjected to an equally satisfactory examination.—*Pacific Medical Journal.*

